# Second-life battery systems for affordable energy access in Kenyan primary schools

**DOI:** 10.1038/s41598-023-28377-7

**Published:** 2023-01-25

**Authors:** Nisrine Kebir, Alycia Leonard, Michael Downey, Bernie Jones, Khaled Rabie, Sivapriya Mothilal Bhagavathy, Stephanie A. Hirmer

**Affiliations:** 1 Energy and Power Group, University of Oxford, Oxford, UK; 2 Aceleron Limited, Bromsgrove, UK; 3 Smart Villages Research Group, Abingdon, UK; 4 Department of Engineering, Manchester Metropolitan University, Manchester, UK; 5 Power Networks Demonstration Centre, University of Strathclyde, Glasgow, UK

**Keywords:** Energy access, Electronic devices, Climate-change adaptation, Energy grids and networks, Electrical and electronic engineering

## Abstract

As the world transitions to net zero, energy storage is becoming increasingly important for applications such as electric vehicles, mini-grids, and utility-scale grid stability. The growing demand for storage will constrain raw battery materials, reduce the availability of new batteries, and increase the rate of battery retirement. As retired batteries are difficult to recycle into components, to avoid huge amounts of battery waste, reuse and repurposing options are needed. In this research, we explore the feasibility of using second-life batteries (which have been retired from their first intended life) and solar photovoltaics to provide affordable energy access to primary schools in Kenya. Based on interviews with 12 East African schools, realistic system sizes were determined with varying solar photovoltaic sizes (5–10 kW in 2.5 kW increments) and lithium-ion battery capacities (5–20 kWh in 5 kWh increments). Each combination was simulated under four scenarios as a sensitivity analysis of battery transportation costs (i.e., whether they are sourced locally or imported). A techno-economic analysis is undertaken to compare new and second-life batteries in the resulting 48 system scenarios in terms of cost and performance. We find that second-life batteries decrease the levelized cost of electricity by 5.6–35.3% in 97.2% of scenarios compared to similar systems with new batteries, and by 41.9–64.5% compared to the cost of the same energy service provided by the utility grid. The systems with the smallest levelized cost of electricity (i.e., 0.11 USD/kWh) use either 7.5 kW or 10 kW of solar with 20 kWh of storage. Across all cases, the payback period is decreased by 8.2–42.9% using second-life batteries compared to new batteries; the system with the smallest payback period (i.e., 2.9 years) uses 5 kW solar and 5 kWh storage. These results show second-life batteries to be viable and cost-competitive compared to new batteries for school electrification in Kenya, providing the same benefits while reducing waste.

## Introduction

To meet growing global energy demands^1^ while combating climate change, much of the world’s present energy use needs to be electrified via renewable energy^2^. As most renewable energy sources, including solar, wind, and tidal energy, are intermittent (i.e., not consistent over time), they must be complemented by energy storage to create systems which can consistently meet energy demands^3,4^.

The need for energy storage applies to both developed and developing countries. In low- and middle-income countries (LMICs), off-grid energy systems containing solar photovoltaics (PV) and batteries are remarkably common, given their plentiful solar resources^5^ and their need to expand energy access to rural areas distant from existing grid infrastructure^6^. Indeed, standalone solar-and-storage systems are increasingly the norm for expanding off-grid energy access in LMICs, and have been shown to provide social, economic, and environmental benefits^7^.

In off-grid energy access applications, lithium-ion batteries are often preferred over lead acid batteries due to their superior energy density and lifetime. Additionally, their ability to be continuously repaired using individual replacement cells^8^ makes them well suited in terms of cost, reliability, and long-term sustainability for remote applications. However, this technology comes with supply chain issues. Lithium-ion batteries are made from rare metals available in limited geographies (e.g., lithium, nickel, and cobalt)^9,10^ which will dwindle in supply as battery demand increases^11^. This is likely to raise prices, making lithium-ion batteries less viable in energy access applications where affordability is essential. It is already difficult to find a sustainable business model for micro- and mini-grids which recovers costs and can be profitable^12^; raising battery prices will exacerbate this situation. Therefore, to meet the demand for lithium-ion batteries at a reasonable cost, efforts must be made to recover, reuse, repurpose and recycle these rare battery materials^13^.

One option to address this is to give batteries a second life after they have reached ‘end-of-life’ in their first use. These are called 'second-life batteries’ (SLBs). Using SLBs instead of new batteries (NBs) can reduce costs and waste while producing reliable renewable energy systems which recover raw materials. This is particularly important given the increasing use of batteries in electric vehicles (EVs), micro- and mini-grids, and utility-scale grid stability^14–17^. It is projected that the global supply of SLBs will reach 112–227 GWh by 2030; this is likely to continue escalating throughout the inevitable large-scale retirement of EVs^18^. It is therefore imperative to find sustainable opportunities to redeploy batteries^19^ as SLBs, given their considerable environmental and economic challenges at end of life^20^. While locally sourced SLBs are likely to be the cheapest option in many cases, there may also be an international SLB market in the near term. The increasing number of retiring batteries in developed countries is likely to raise challenges for those countries to recycle, reuse or repurpose them all internally. Exporting SLBs at low cost to LMICs could therefore become an attractive and mutually beneficial option.

In light of this, this work assesses the feasibility and potential benefits of using hybrid SLB and PV systems in energy access systems for schools in Kenya. While the idea of hybrid PV-battery energy access systems for schools has been discussed in Refs.^21–23^, these studies did not consider SLBs. The use of SLBs for rural households' electrification to improve general living conditions has been discussed in Refs.^24,25^; however, the idea of incorporating SLBs coupled with PV systems in rural schools proposed in the current study is novel. The techno-economic feasibility of such systems requires study to ensure that they can lead to increased and enhanced energy access at a competitive cost, making them a viable use case for end-of-life batteries.

We consider the use of SLBs coupled with PV in Kenyan primary schools. This was informed by semi-structured interviews with stakeholders and members of staff in 12 East African schools which elicited energy needs, aspirations, and technical and financial challenges. The energy characteristics of four of these schools in Kenya are studied in depth. We take one as a representative case study for techno-economic analysis, evaluating the costs and reliability of providing energy using (1) systems containing SLBs, (2) systems containing NBs, and (3) grid-connected energy. An optimal system size and type to implement is proposed using realistic assumptions from the wider literature. Considering these results, we draw conclusions on the feasibility of SLBs in this application to reduce battery waste while creating cross-cutting benefits. Note that all assumptions related to lithium-ion batteries in this paper are specifically for lithium nickel manganese cobalt oxides.

### Second-life battery uses

When discussing SLBs, it is important to distinguish between reuse, repurposing, and recycling. Whereas reuse involves directly using a material again for the same purpose, repurposing gives a material a new purpose than for what it was originally intended, and recycling involves breaking down the product to a more fundamental level for reuse of constituent components. When considering SLBs, battery reuse or repurposing often maximises value compared to recycling, as these options consume less energy^26^. Most batteries are currently assembled without a recycling focus, meaning that certain parts—particularly the cells that contain valuable metals—are permanently connected by welding and/or glue, making them difficult to extract. As such, applications that can reuse or repurpose batteries without disassembly are likely to be the most cost effective for current SLB stock.

Lithium-ion batteries from electric vehicles (EVs) are particularly high-value SLBs. At their ‘end-of-life’, they still typically have 70–80% of their capacity remaining^27^. This equates to thousands of charge/discharge cycles and hours of usable energy storage. While these batteries may no longer be appropriate for use in EVs, there are many other applications which can make valuable use of this remaining capacity, as reviewed by a number of other authors^20,28,29^. Based on the literature and author field experience, SLB applications of interest in LMIC contexts include light mobility, single-building power supplies, and micro- and mini-grids; the suitability of SLBs in these cases is elaborated in Table 1. One promising opportunity for SLB redeployment is to enable affordable electricity access in school settings. This falls into the single-building power supply category listed in Table 1, which is the focus of this research, as we investigate this repurposing potential for primary schools in Kenya.

**Table 1:** Applications of interest for lithium-ion second-life batteries from electric vehicles in low- and middle-income countries.

Application	Suitability to lithium-ion second-life batteries	Battery capacity at end of second life
Light mobility (e.g., scooters, quad bikes)	Do not require long-distance range; high-end power and intense cycling requirements. However, these vehicles are smaller than EVs so may be difficult to retrofit in SLBs from EVs; high demands on weight/power density	40–60% (highly dependent on vehicle and usage)
Single building power supply	More flexible size and weight requirements than light mobility; often less taxing power requirements; often not the sole and critical power supply point (e.g., house also has solar PV or grid connection)	35–40%
Micro- and mini-grids	Least sensitive to weight and space (i.e., since housed in a central, purpose-built facility); can use larger numbers of lower capacity batteries to be combined in series to provide the desired capacity for the system	35%

### Opportunities for second-life batteries in school energy access

There are approximately 32,437 primary schools in Kenya. According to a government spokesperson, in December 2017, 76% of these schools had access to electricity^30^. However, even where schools are connected to the grid, power is unreliable^31^. This means that pupils are often forced to limit their learning to daylight hours, or to when power is available. Electricity outages also limit access to modern educational technologies (e.g., computers, projectors) which, while not an educational panacea, play a complementary role and fill instructional gaps^32^. Not being able to use these technologies can disrupt lesson plans and limit consequent learning.

Given the resource constraints in Kenya’s educational system, the high costs of using grid electricity compete with other critical school expenses. A 2019 study of 300 boarding schools in Kenya showed an average monthly spend of $4000 on electricity alone^33^; this is equivalent to the average cost of employing two teachers in the Nairobi area^34^. As such, with limited budgets available, schools may be forced to cut costs by employing fewer teachers or minimising electricity use, each of which negatively impacts learning.

Alternative and complementary off-grid energy sources for schools, such as solar PV systems with batteries, can offer a more cost effective and reliable energy alternative. Indeed, the use of hybrid systems for electrification has shown benefits in East Africa, and for schools in particular which rely on lighting and information technologies to provide quality education^21,35,36^. However, particularly in poorer rural communities, there is often still a lower willingness and ability to pay for such systems^37^; as such, costs need to be minimised wherever possible. The reuse of lithium-ion batteries could have a significant impact on recovering some of the costs involved in building micro-grids or off-grid systems^25^, passing affordability benefits onto the consumer.

There are also numerous waste management challenges associated with standalone systems, as previously discussed^38^. To illustrate, around 700 tonnes of solar e-waste were discarded in Kenya alone in 2016^39^. When discarded improperly, batteries can cause major health concerns to nearby communities^40^; as such, reducing their wastage where possible is critical. By instead repurposing and re-using these batteries as SLBs in off-grid systems, we hypothesise that affordable systems can be designed for school electrification which also reduce waste, avoid health harms, and provide social benefit.

## Results

### Energy needs and challenges assessment

An energy needs assessment was undertaken in 12 schools (eight in Kenya and two each in Uganda and Tanzania) via semi-structured interviews. These were selected on the basis of existing contacts and data access. Interviews covered: current energy use (including devices and hours of use), sources, costs, and satisfaction; energy use aspirations (i.e., what devices they would connect if energy were more abundant, reliable and/or affordable); whether they would consider an alternative source of energy, especially from a solar/battery system; and demographics.

The principal uses of electricity identified in the interviews were classroom lighting, security lighting, information technology (e.g., computers, printers/copiers, projectors, etc.), and phone charging. These all use relatively little power, which speaks well to the potential suitability of hybrid battery and PV solutions to meet school energy needs. It was reported that these technologies impacted the ability of students to study (lighting), the security of the school community and premises (security lighting), the ability of teachers to prepare and deliver lessons (phone charging, laptops), preparation for examinations (printing and copying), and basic life/livelihood skills for better educational outcomes (information technology labs). Some aspirational productive uses of electricity were also identified by interviewees (e.g., submersible borehole pumps for water, refrigeration for food preservation). However, these are considerably more challenging to power from a hybrid battery and PV system and are also less core to the learning purpose of the school. As such, these high-powered devices were not carried forward into the techno-economic analysis.

Interviewees at grid-connected schools reported technical and financial challenges in using electricity. They reported that power was unreliable due to frequent and prolonged network outages. They also reported that expensive utility electricity contributed to schools’ high costs. This, combined with a lack of (or late payment of) funds to cover school bills from central funding sources, meant that interviewed schools often had to seek additional financial support from pupils’ parents to be able to pay their electricity bills. This indicates an urgent need to provide an affordable and reliable energy access alternative. The impact of schools having limited, expensive electricity was reported to result in reduced educational outcomes, increased financial burden for parents, poor staff retention, and ultimately reduced school attendance.

### Basic characteristics of case study schools

Of the sampled schools, four schools in Kenya were characterised in more detail: two situated in Nairobi, and one each in Kagiado and Machakos counties, as listed in Table 2. Schools 1, 2, and 3 are grid connected, while School 4 is not electrified at present and uses a stand-alone biogas plant for school cooking needs. Schools 1 and 2 are urban while Schools 3 and 4 are rural. They are all state schools supported by the government.

**Table 2:** Characteristics of the four Kenyan primary schools selected for detailed study.

School	County	Urban or rural	Average # of students (2021)	Annual utility cost (USD)	Maximum load (kW)	Daily device usage (h)
1	Nairobi	Urban	158	$2310	4.290	24
2	Nairobi	Urban	150	$1638	5.955	2–10
3	Kagiado	Rural	468	$1311	3.625	5–12
4	Machakos	Rural	600	$0	6.075	8–10^a^

^a^School 4 uses devices like laptops but charges them externally, since they are not grid connected and do not have off-grid electrical power.

Drawing from the needs assessment, Table 2 reports the average number of students per school, their required hours of electricity use, the maximum aspirational load of each school, and their annual electric bills. Note that School 1 uses electricity throughout all hours of the day as there was a reported need to provide continuous low lighting for security reasons at night.

**Figure 1:**
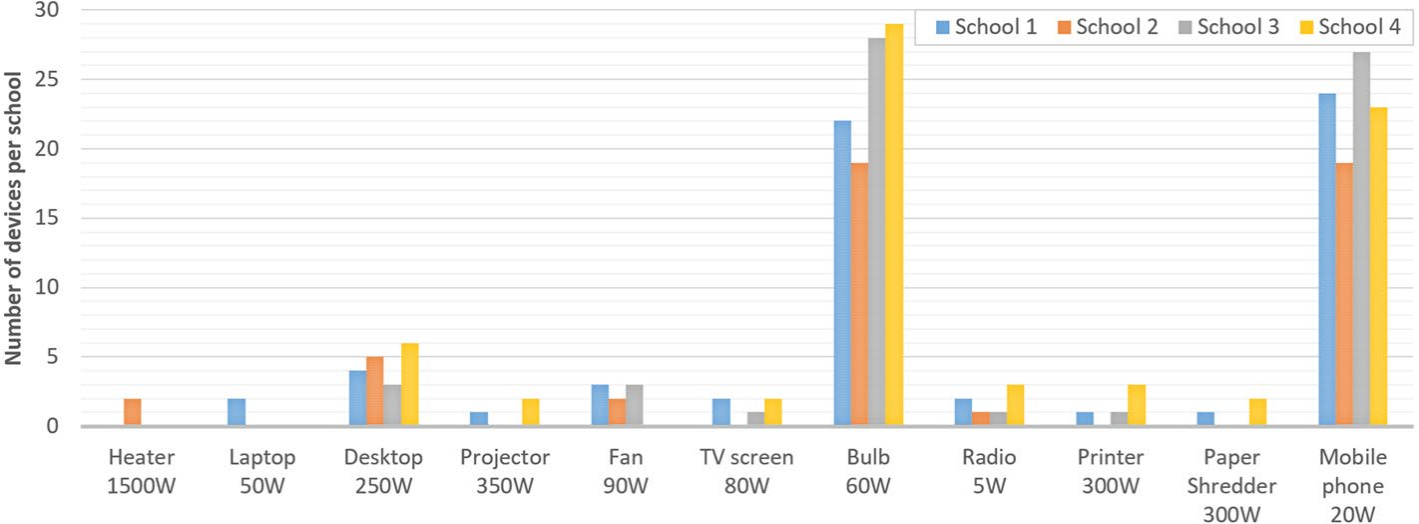
Quantity of each appliance type desired per school, and their rated power in watts (W).

The maximum aspirational load of these schools shown in Table 2 reflects the distribution of electrical appliances desired by the four schools, as illustrated in Fig. 1. These appliances are roughly the same for the four schools and require an average installed power around 5 kW. Based on each school’s appliance aspirations, projected loads were relatively consistent during school opening days during term time, and less (but not zero) during weekends and holiday closure. The most marked seasonal variation noted within projected demand was a greater use of printing and copying in the lead up to the examination period. This allows students to do practice tests and allows schools to provide students with revision material.

### Techno-economic analysis of second-life battery system feasibility

We next conducted a techno-economic analysis to assess the impact of introducing hybrid PV and battery systems in these schools. Both NB and SLB options were studied, and outcomes compared in terms of capital cost, transportation cost, energy reliability, and dependency on grid supply to supplement the system. While we primarily focus on the comparison of locally sourced SLBs with imported NBs, as a sensitivity analysis, we also assess the economic impact of using imported SLBs in the event of local unavailability. We also investigate potential opportunities stemming from using imported SLBs at a low capital expenditure (CAPEX) cost. Therefore, we examine 48 scenarios including different combinations of PV, NBs, SLBs, assuming that NBs are always imported and SLB might either be local, imported at average CAPEX, or imported at a minimum CAPEX (i.e., 0.04 USD/kWh^41^). Given the relative similarity of the four characterised schools, we focus on School 1 as a representative case study for this analysis.

We calculated the hourly annual load demand curve of the school based on the appliance aspirations resulting from the needs assessment (for a fuller description, see “Methods”). This resulted in an annual average energy need of 10,220 kWh. We then calculated the potential solar PV production using a set of PV sizes ranging from 5 to 10 kW with an increment of 2.5 kW. Similarly, the storage capacity was selected from lithium-ion battery sizes ranging from 5 to 20 kWh with an increment of 5 kWh. These sizes are selected as they are the most common available solar system sizes^42^ and battery sizes in the region. As such, they are most likely to be available locally as retired NBs, which are available to be redeployed as SLBs to help provide energy access in schools.

**Figure 2:**
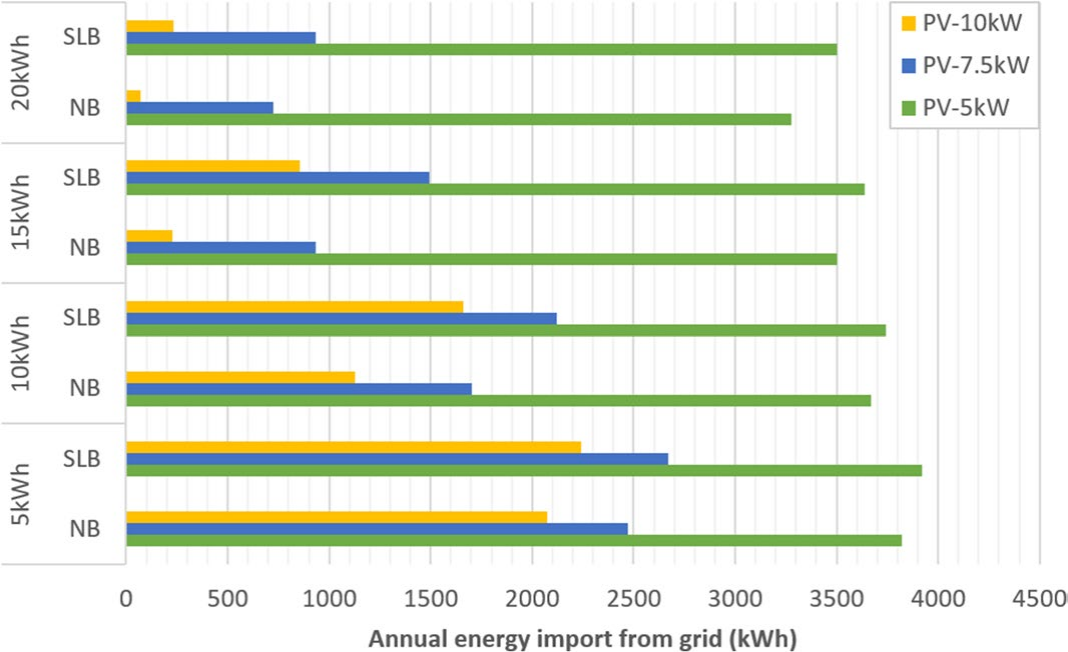
Energy imported from the grid per year in each system size and battery type scenario, considering second-life batteries (SLB) and new batteries (NB).

We studied the annual import of energy from the grid required by School 1 in kWh for each scenario. The results (see Fig. 2) show that, as expected, dependency on the grid reduces with increased energy system capacity. However, grid dependency increases when SLBs are used compared to NBs. This makes sense, as grid dependency is impacted by the technical performances of the batteries in terms of (1) state of charge, which is reduced for SLBs compared to NBs, and (2) self-discharge rate, which is higher for ageing batteries (see Table 3). Considering that grid reliability is already an issue in schools, reducing grid dependency in terms of the number of days is key, particularly as outages increase in the region^35,36^.

**Table 3:** Economic and technical assumptions for techno-economic analysis.

Parameter	New batteries	Second-life batteries	Source
SOC minimum	10%	20%	^43^
SOC maximum	90%	80%	^43^
Self-discharge rate	0.001%	0.01%	^43^
Lifetime (years)	13	5	^44^
Inflation	5.5%		^45^
Discount rate	5%		^46^
Annual PV degradation	0.5%		^46^
Annual battery degradation	2.0%	2.6%	^47^
Cost of batteries ($/Wh)	0.283	0.040–0.096	^41,48^
Cost of PV ($/W)	1		^49^
Battery O&M cost ($/kWh)	1.16	2.17	^50,51^
PV O&M cost ($/kW)	9		^52^
O&M escalator	2%		^53^
Transportation cost ($/kWh)	28.02		^54^

**Figure 3:**
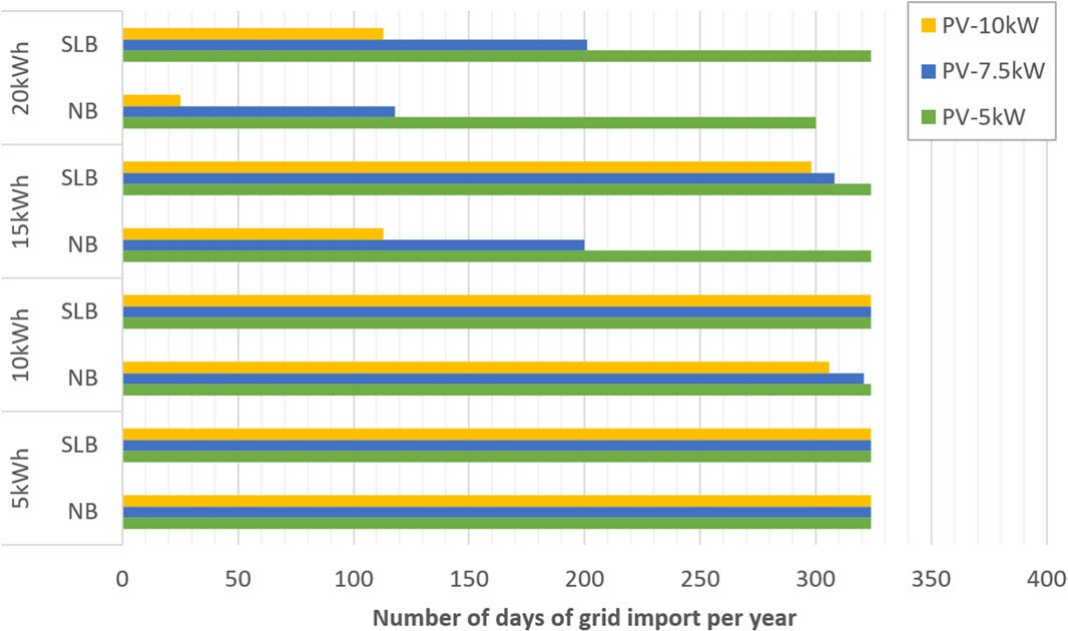
Number of grid-dependent days per year in each system size and battery type scenario, considering second-life batteries (SLB) and new batteries (NB).

Figure 3 illustrates the number of days the grid is needed to provide energy to School 1 for each scenario. This directly correlates to the capacity of the battery in the scenario and how many hours this could cover in the daily demand peak of the year. This varies between 20 and 100% for both energy storage system types studied here. These results show that the yearly dependency on the grid could be reduced to 25 days for the NB case and 113 days for the SLB case, in the 10 kW PV and 20 kWh storage capacity scenario.

**Figure 4:**
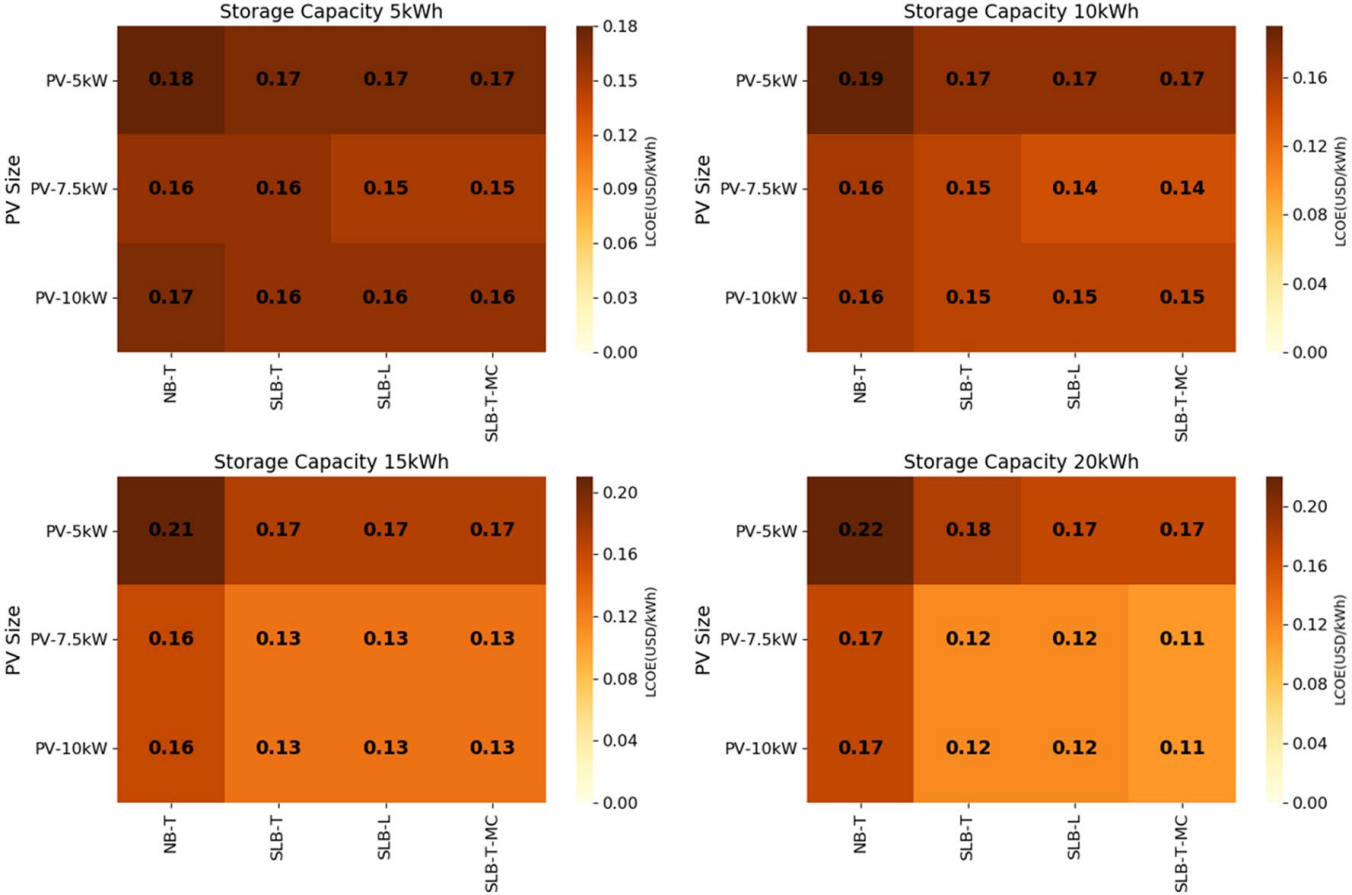
Levelized cost of energy (LCOE) per scenario, including options with new batteries transported (NB-T), second-life batteries transported (SLB-T), local second-life batteries (SLB-L), and second-life batteries transported at minimum CAPEX (SLB-T-MC). Across the 12 system size options, SLBs reduce LCOE compared to NBs in almost all of the cases studied.

To assess the impact of each hybrid scenario on the school’s electricity cost, we compared the levelized cost of electricity (LCOE) in each scenario over 25 years with the LCOE calculated from the utility. This was estimated at 0.31 USD/kWh, resulting from the current electricity cost of 0.28 USD/kWh and considering an annual utility escalating factor of 1%. Results for each scenario show that the LCOE varies between 0.11 USD/kWh and 0.22 USD/kWh (see Fig. 4) and that in 97.2% of the scenarios studies, using SLBs is cheaper compared to NBs as per the LCOE obtained. This was calculated using the methodology described in the “Methods” section and the assumption on Table 3 and matches results on LCOE for NB in hybrid systems in Kenya from previous studies on a discount rate basis of 5%^46^. The various scenarios for LCOE calculation have included the impact of the NB and SLB replacement over 25 years (see Table 3) such as the operations and maintenance cost increase and the battery efficiency improvement following the replacement.

**Figure 5:**
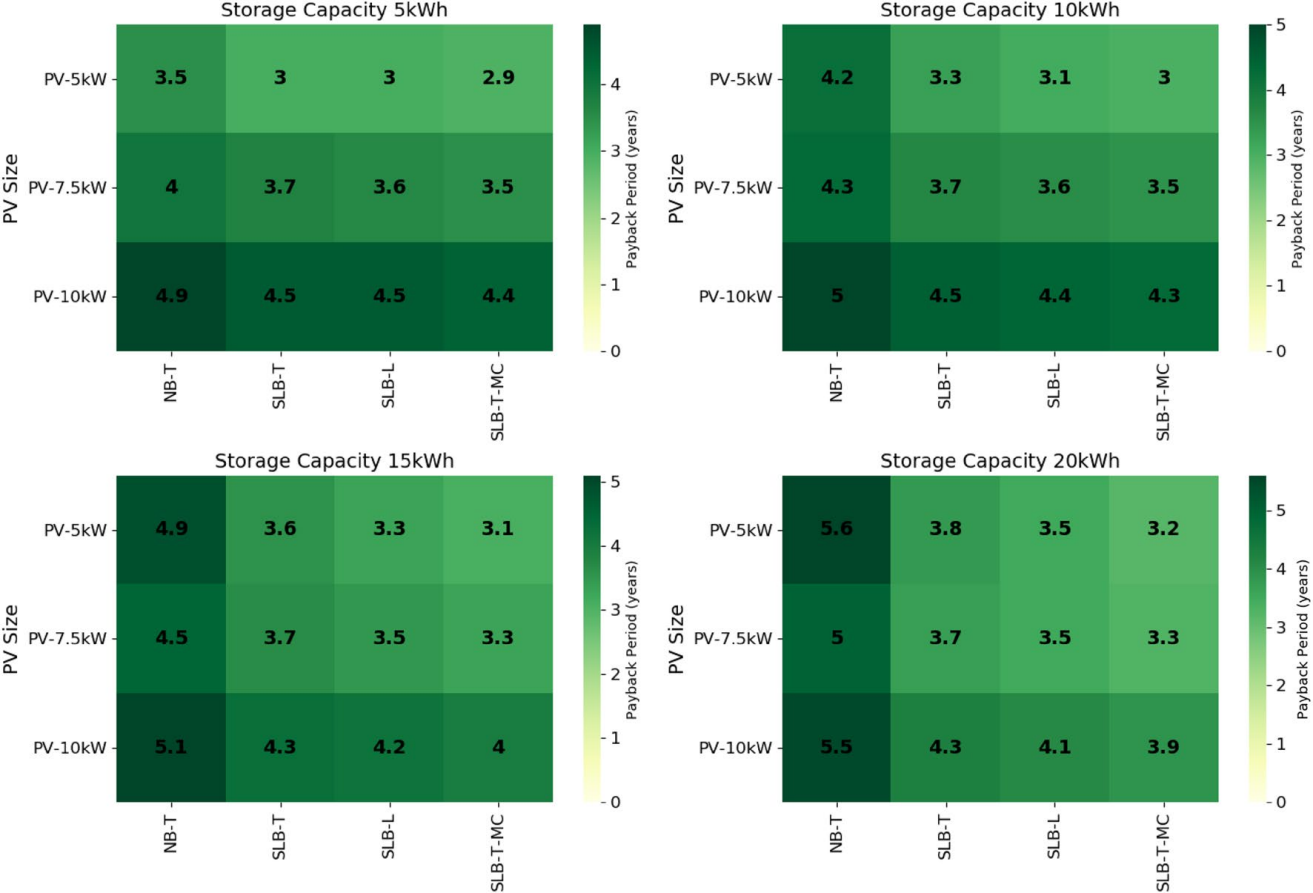
Payback period for each scenario, including options with new batteries transported (NB-T), second-life batteries transported (SLB-T), local second-life batteries (SLB-L), and second-life batteries transported at minimum CAPEX (SLB-T-MC). The system with 5 kW PV and 5 kWh SLB has the shortest payback period.

The cost benefit of switching from grid connected electricity to one of the hybrid SLB or NB systems specified here is studied in terms of the system payback period (PBP). The results, illustrated in Fig. 5, show that the minimum PBP is 2.9 years, corresponding to the use of a hybrid system composed of 5 kW PV and 5 kWh SLB storage.

## Discussion

This work shows the potential benefits of using hybrid PV and battery systems containing lithium-ion SLBs in Kenyan schools. The enhanced lifespan and ease of repair of these batteries positions them well to offer cheaper, reliable, and more sustainable electricity access than NB alternatives; their economic and practical viability is proven for the case of school electrification in this work.

We have shown that the use of repurposed lithium-ion SLBs provide huge affordability benefits to the end user of energy systems compared to NBs. This agrees with other studies that used SLBs in similar applications (e.g., residential usage in Ref.^42^, mini-grid in Tanzania in Ref.^24^). In fact, locally sourced SLBs are cheaper than imported NBs in all of the scenarios studied, with a maximum reduction in LCOE (29.4%) for the (1) 7.5 kW PV and 20 kWh SLB storage, and (2) 10 kW PV and 20 kWh SLB storage systems. The latter reduction in LCOE has also been identified for the use of imported SLBs in the systems (1) and (2); however, it was found that importing SLBs over using local SLBs in the systems (3) 7.5 kW PV and 5 kWh SLB storage, (4) 7.5 kW PV and 10 kWh SLB storage, and (5) 5 kW PV and 20 kWh SLB storage is not economically encouraged.

The sensitivity analysis undertaken here shows that low-CAPEX SLB imports are an exciting opportunity in LMICs in the near term. The results for the low expense imported SLB case (SLB-T-MC) show an LCOE drop of 5.6–35.3% in all scenarios compared to NBs. This is a 16.7% drop compared to local or imported SLBs at average market cost for the same preferred systems. Therefore, while the international transportation of lithium-ion batteries can be costly (i.e., around 28.02 $/kWh^54^; the import duties of lithium-ion account for up to 35% of the cost of the product in Kenya^55^) and subject to complex regulations^56^ (e.g., due to their perception as dangerous based on previous incidents where they have caused fires^61^; furthermore, international transport regulations can class SLBs as waste, which is subject to different regulations than consumer goods (e.g., Ref.^62^ in the United Kingdom)), there are still opportunities in using imported batteries compared to NBs. If international transit difficulties can be overcome, using SLB originating from EVs in developed countries^56^ for hybrid systems in Kenya would be a low-cost opportunity to reduce waste and at the same time enhance energy access. Until the EV sector is established in Kenya, or the second-hand EV market begins to flourish, SLB import is therefore a viable option. As the stock of locally available SLBs increases (e.g., as EVs are increasingly used domestically), the local market is likely to undercut these low-CAPEX imports, as they can avoid custom costs and duties. These local SLBs will create new value from otherwise waste-bound batteries without the need to cross borders, reducing costs for end users.

Regarding the payback periods calculated, we find that schools will be able to pay back hybrid system costs in a shorter period of time when SLBs are used compared to NBs in all of the scenarios studied. The shortest system payback period was found to be 2.9 years for the 5 kW PV and 5 kWh SLB scenario. Beyond these cost benefits, we have also found that using SLB systems in schools can decrease grid dependency. This is important given the high number of network outages that occur in rural and urban areas of Kenya, which are greatly disruptive to learning.

While we have shown that SLBs can reduce the costs of school energy access in Kenya significantly while effectively mitigating the challenges of battery waste, there are still a number of issues that need to be overcome for SLBs to be effectively used on a wide scale. These are highlighted in the following opportunities for further research and development.

- **There are still limited end of life strategies for SLBs**. SLBs of course bring benefits in terms of waste management of NBs, which are considered to be one of the key hazardous wastes to be reduced in sub-Saharan Africa^57^. However, SLB systems are not a permanent waste management solution. SLBs will also, eventually, reach the end of their second life. At this point, recycling their materials is likely to be the most economic and sustainable remaining waste management option. There is therefore still a need to research and develop better battery recycling strategies to fulfil this future need^27^.
- **SLBs require monitoring and control, which can be difficult in remote areas**. Monitoring of SLBs is needed to schedule preventive and predictive maintenance, which is liable to be somewhat less predictable than that of NBs given their varying first-life uses. It is also needed to facilitate resource sharing amongst batteries, loads, and PV in unified off-grid systems. In rural areas, which as previously discussed are good candidates for off-grid SLB-based electrification, this monitoring needs to be done remotely to keep costs down, which is challenging. However, in a country like Kenya which places a high importance on mobile connectivity (e.g., as evidenced by its largely mobile-based financial system M-PESA^58^), there is an opportunity for internet of things (IoT) sensors combined with mobile connectivity to play this monitoring role. IoT can be used to collect important system parameters (e.g., state of charge, number of cycles) for more efficient utilisation of available devices (e.g., batteries, solar panels and loads). More specifically, narrowband IoT has a great potential to further improve the performance and reliability of SLB energy access systems^59^. This could facilitate (1) resource sharing within a community or school system, providing further benefit from SLBs at minimal infrastructure cost, (2) optimised performance and maximised energy efficiency and sustainability, and (3) in the case studied here, prolonged time spent at school and consequent educational benefit. Motivated by the above, as future work, the authors intend to practically investigate the achievable gains that can be obtained with the IoT-equipped SLB system.
- **Lack of awareness of the benefits of SLBs**. Despite these significant potential benefits of SLBs, there are still barriers to their adoption, including technology awareness. For the benefits of lithium-ion SLBs to be realised, appropriate knowledge sharing needs to be undertaken with regards to the benefits of lithium-ion SLBs to increase the likelihood of adoption. In this work, a second round of interviews conducted with the same 12 East African schools pointed to a gap in knowledge of the benefits of SLBs. Stakeholders expressed a reluctance to buy into the idea of SLBs as opposed to NBs. To promote SLB uptake in this case, the educational institution would ideally be leveraged to raise awareness and train the community members to maintain the SLB system. In a similar vein, future work should also explore the potential of sustainable battery centres which build, service, maintain, and recycle end-of-life NBs situated near schools or facilities using the SLB systems. This could provide local operations and maintenance expertise and also be an economic opportunity to promote clean development.

## Methods

### Interview data

This work uses semi-structured interview data obtained through partners in anonymized aggregate to determine school energy use, challenges, and aspirations from a representative sample. Data from 12 primary schools is used—eight in Kenya, and two each in Tanzania and Uganda. Interviews were carried out by partners with either the head teacher or the administrative manager of the school. These interviews were coordinated by Smart Villages Research Group Ltd. and undertaken by three partner organisations based in-country whose normal activities are not in education or school energy provision, making them neutral parties for data collection. Schools were made aware that there was no expectation that they would be provided with any off-grid energy technology or solutions to support their energy needs, with an aim to eliminate artificially optimistic or aspirational responses.

Interview results were only shared with the research team in anonymized aggregate. The interview process underwent ethical review through the Smart Villages Research Group Ltd. ethics committee, which consists of the principal staff and advisors, and received full approval. All methods were performed in accordance with the relevant guidelines and regulations, including informed consent, which was obtained for all interviews.

A first round of interviews was carried out in April and May of 2021, when many schools were just emerging from lockdowns. Face-to-face visits were therefore often not feasible, so part of the selection criteria for the schools was the availability of a senior local contact, who could be phoned and would be knowledgeable regarding school activities, economics, and practices. The questions in the first semi-structured interview included: current energy use (including devices and hours of use), sources of energy, average costs, and satisfaction; energy use aspirations (i.e., what devices they would connect if energy were more abundant, reliable and/or affordable); whether they would consider an alternative source of energy, especially from a solar/battery system; and demographics. A second round of interviews was then completed in March 2022 with the same schools to present them with some of the conclusions of the techno-economic analysis (e.g., some sample system costings, and estimated grid-savings and reduction of reliance on the grid) and to ask them their reactions to the options. They were asked whether any of those might be attractive to their school, and about their general understanding of batteries in solar power systems, lithium-Ion batteries, and SLBs in particular.

### Data analysis

#### Electricity demand forecast and yearly load profile

The daily load profile, on an hourly timescale, through the year for School 1 was calculated based on the following assumptions:

- The electrical appliances used and desired in the school, their rating and number of hours of use per day as provided by the school staff (see Table 2 and Fig. 1):
  - Fans turn-off in the rainy season (the long rainy season lasts from March to May, while the short rainy season lasts from October to November).
  - Printers on four hours during terms and eight hours in exams periods.
- The school terms and holidays scheduled between 2021 and 2023, to build an average yearly figure (see Table 4):
  - School days are Monday–Saturday.
  - The term dates are always January to April, May to August and September to November.

**Table 4:** Kenya school terms and holidays periods (2021–2023).

	2021 Kenya School Calendar		2022 Kenya School Calendar		2023 Kenya School Calendar	
	Period	Duration	Period	Duration	Period	Duration
Term 1	26/07/2021–01/10/2021	10 weeks	25/04/2022–01/07/2022	10 weeks	23/01/2023 21/04/2023	13 weeks
Half term break	26/08/2021–29/08/2021	3 days	26/05/2022–29/05/2022	3 days	23/03/2023 26/03/2023	3 days
Holiday	02/10/2021–10/10/2021	1 week	2/07/2022–10/07/2022	1 week	22/04/2023 07/05/2023	2 weeks
Term 2	11/10/2021–23/12/2021	11 weeks	11/07/2022–16/09/2022	10 weeks	08/05/2023 11/08/2023	13 weeks
Christmas break	24/12/2021–2/01/2022	10 days	11/08/2022–14/08/2022 17/09/2022–25/09/2022	3 days and 1 week	29/06/2023–02/07/2023 12/08/2023–27/08/2023	3 days and 2 weeks
Term 3	03/01/2022–04/03/2022	9 weeks	26/09/2022–25/11/2022	9 weeks	28/08/2023 03/11/2023	10 weeks
Primary examinations (KCPE)	07/03/2022–10/03/2022	4 days	28/11/2022–1/12/2022	4 days	06/11/2023 09/11/2023	4 days
Primary examinations (KCSE)	11/03/2022–01/04/2022	3 weeks and 1 day	01/12/2022–23/12/2022	3 weeks and 1 day	10/11/2023 01/12/2023	3 weeks and 1 day
KCSE marking	04/04/2022–22/04/2022	3 weeks	02/01/2023–20/01/2023	3 weeks	04/12/2023 22/12/2023	3 weeks

### Solar PV production modelling

The annual electricity generated by the PV systems for the various sizing on an hourly basis through year 2019 (as the year of reference), was calculated using a combination of inputs:

- Expected yield factor from solar array (2019 data) are taken from the https://www.renewables.ninja/ website.
- Capacity of the PV array for each scenario.

### Assessing electrification costs

To compare the utility cost to the grid-connected hybrid systems proposed including PV arrays and battery energy storage systems, we have used the following LCOE (USD per kWh) formula based on the Net Present Values (NPV) calculation^46^:

$$LCOE= \frac{{C}_{0}+\sum_{t=1}^{N}\frac{{C}_{PV+Storage+Grid}^{t}}{{(1+r)}^{t}}}{\sum_{t=1}^{N}\frac{{E}_{PV+Storage+Grid}^{t}}{{(1+r)}^{t}}}.$$

${C}_{0}$ represents the capital cost initially invested (including transportation costs if relevant depending on the scenario studied), ${C}_{PV+Storage+Grid}$ and ${E}_{PV+Storage+Grid}$ are the yearly costs and energy produced by the hybrid system over the lifetime of the project ($N$), and $r$ is the discount rate. This formula applies for both NBs and SLBs.

To calculate the number of years the school will need to recover what has been invested, the payback period has also been calculated for each scenario by dividing the investment cost by the yearly cash flow^60^.

## Data Availability

Raw interview data collected to inform the current study are not publicly available to protect the privacy and anonymity of the schools involved. The research team only accessed these data in anonymized aggregate. The aggregated results which are pertinent to the analysis presented here are shown in-text in Table 2 and Fig. 1. Further details are available from the corresponding author on reasonable request.
